# 1017. Impact of a Multifaceted Approach to Audit-and-Feedback Antimicrobial Stewardship Intervention in Acute Leukemia Patients Using Measures of Appropriateness

**DOI:** 10.1093/ofid/ofad500.048

**Published:** 2023-11-27

**Authors:** Miranda So, Ghadeer Alahmadi, Bassem Hamandi, Shahid Husain

**Affiliations:** University Health Network, Mississauga, ON, Canada; University Health Network, Mississauga, ON, Canada; University Health Network, Mississauga, ON, Canada; University Health Network, University of Toronto, Toronto, ON, Canada

## Abstract

**Background:**

Prospective audit-and-feedback (PAF) for hospitalized leukemia patients is resource-intensive, while data on stadardized assessment of the quality, i.e. appropriateness, of antimicrobial prescribing are scarce. We implemented a multifaceted program combining patient-level PAF with unit-level appropriateness reports generated by serial point-prevalence surveys using the web-based Canadian National Antimicrobial Prescribing Survey (NAPS^TM^) platform. Quality of prescribing was assessed based on local guidelines and clinical justification using published definitions (Fig. 1). The objective of the study was to evaluate the impact of PAF+NAPS interventions on antibiotic use (AU) in acute leukemia patients at an academic cancer center, compared to PAF-only.

**Figure:**
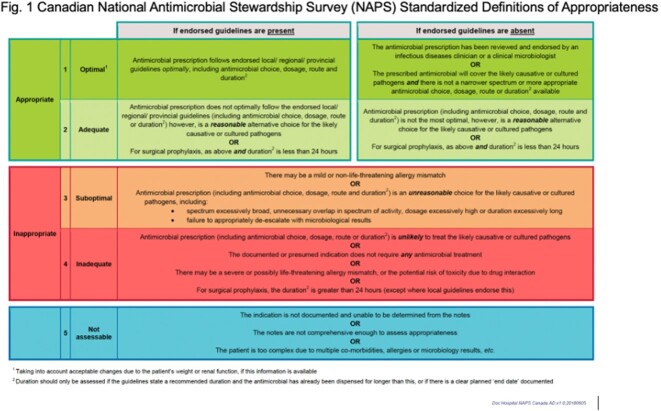

**Methods:**

In this retrospective cohort study, during the PAF-only period (Jun 1 2017-May 31 2019), the AMS team led 30-minute PAF rounds with prescribers 2x/week. After implementating PAF+NAPS (Jun 1 2019-Mar 31 2020), we met 2x/month for 30 minutes. At each meeting we presented an automated NAPS dashboard report (Fig. 2) of aggregate appropriateness adjudication of antibiotic prescriptions, followed by targeted PAF on select patients with complex antimicrobial needs. We analyzed the impact of the two-step approach on the primary outcome of AU (defined daily dose [DDD]/100 patient-days [PD]) using interrupted time series models. Secondary outcomes were appropriateness of AU during PAF+NAPS, lenght of stay and in-hospital mortality. Target appropriatenesss was at least 90%. Categorical variables were compared using chi-square test and continuous variable using Mann Whitney U test.

**Fig. 2:**
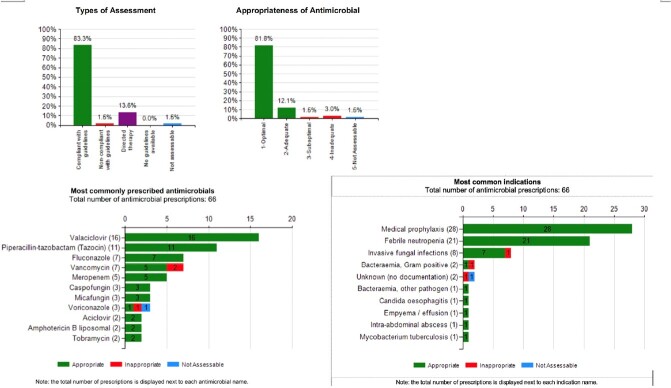
Sample Canadian NAPS Dashboard Report From a Point-Prevalence Survey

**Results:**

Patient characteristics and outcomes were presented in Table 1. AU data were presented in Table 2. AU of piperacillin-tazobactam and vancomycin decreased significantly after implementation of 2x/month PAF+NAPS, compared to 2x/week PAF-only interventions (Fig. 3). Overall appropriateness was 91.4% (1757/1921 prescriptions).

**Figure:**
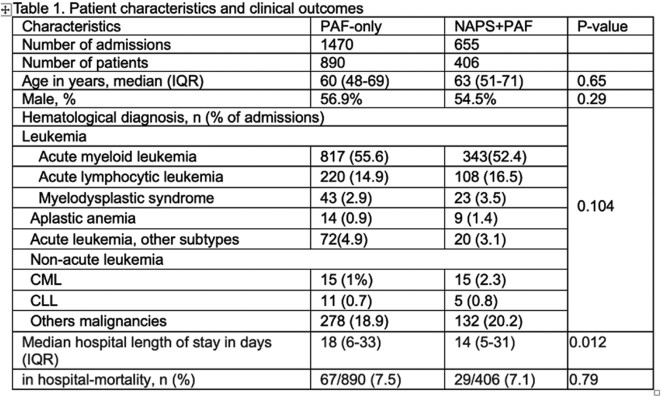

**Figure:**
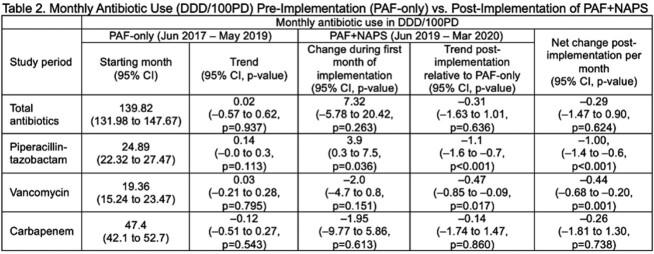

**Figure:**
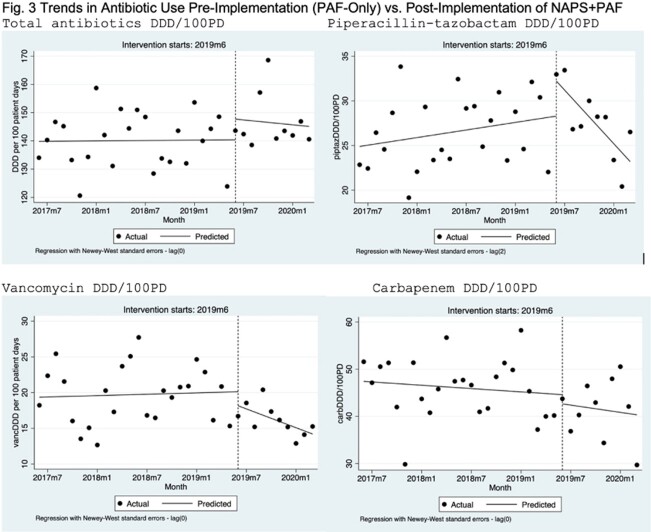

**Conclusion:**

Despite reducing frequency of interventions from 2x/week to 2x/month, PAF+NAPS was an efficient and sustainable AMS model that promoted appropriate and judicious antimicrobial use in hematology-oncology patients (Fig. 4), allowing the AMS team to expand to other specialized areas.

**Figure:**
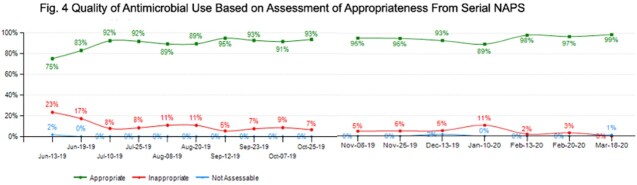

**Disclosures:**

**Shahid Husain, MD**, Astellas: Grant/Research Support|Avir: Grant/Research Support|F2G: Grant/Research Support|Gilead: Grant/Research Support|Merck: Grant/Research Support|Pfizer: Grant/Research Support|Pulmocide: Grant/Research Support|Synexis: Grant/Research Support|Takeda: Honoraria

